# Training and Evaluation of Human Cardiorespiratory Endurance Based on a Fuzzy Algorithm

**DOI:** 10.3390/ijerph16132390

**Published:** 2019-07-05

**Authors:** Jui-Chuan Cheng, Chao-Yuan Chiu, Te-Jen Su

**Affiliations:** 1 Department of Electronic Engineering, National Kaohsiung University of Science and Technology, Kaohsiung 80782, Taiwan; 2 Department of Biomedical Engineering, Kaohsiung Medical University, Kaohsiung 80708, Taiwan

**Keywords:** fuzzy algorithm, cardiorespiratory endurance, resting heart rate, heart rate recovery, IoT

## Abstract

Cardiorespiratory endurance refers to the ability of the heart and lungs to deliver oxygen to working muscles during continuous physical activity, which is an important indicator of physical health. Cardiorespiratory endurance is typically measured in the laboratory by maximum oxygen uptake (VO_2max_) which is not a practical method for real-life use. Given the relative difficulty in measuring oxygen consumption directly, we can estimate cardiorespiratory endurance on the basis of heart beat. In this paper, we proposed a fuzzy system based on the human heart rate to provide an effective cardiorespiratory endurance training program and the evaluation of cardiorespiratory endurance levels. Trainers can respond correctly with the help of a smart fitness app to obtain the desired training results and prevent undesirable events such as under-training or over-training. The fuzzy algorithm, which is built for the Android mobile phone operating system receives the resting heart rate (RHR) of the participants via Bluetooth before exercise to determine the suitable training speed mode of a treadmill for the individual. The computer-based fuzzy program takes RHR and heart rate recovery (HRR) after exercise as inputs to calculate the cardiorespiratory endurance level. The experimental results show that after 8 weeks of exercise training, the RHR decreased by an average of 11%, the HRR increased by 51.5%, and the cardiorespiratory endurance evaluation level was also improved. The proposed system can be combined with other methods for fitness instructors to design a training program that is more suitable for individuals.

## 1. Introduction

### 1.1. Cardiorespiratory Endurance

With the rapid development of the economy, manual labor work has been replaced by a large number of machines, resulting in an inactive state of life. The World Health Organization points out that inadequate physical activity has become the fourth most important risk factor for global mortality, more than 2 million deaths can be attributed to physical inactivity. About 60–85% of adults in the world live a static life, and two-thirds of children have insufficient physical activity, which will affect their health and cause public health problems in the future [1,2]. From a health perspective, people who have good heart and lung endurance can exercise longer, not get tired as quickly, and avoid all kinds of cardiorespiratory diseases. Improving cardiorespiratory endurance is an important issue for maintaining good health [3,4,5].

To eliminate the need to refer to a chart of the modular standard to decide the fitness category, A back-propagation neural network (BPNN) was adopted to accesses the subject’s physical fitness (PF). Data collected included five parameters required for the PF passport: subject’s age, body mass index (BMI), performance in the sit-and-reach test, 1-min bent-leg curl-ups, and cardiorespiratory endurance [6]. 

The appropriate exercise level varies for each elderly person because there are great individual differences. To remain healthy by exercising, and to provide appropriate exercise levels for elderly people, a fuzzy system is designed for adjusting the cycle ergometer workload to each individual’s physical work capacity. For the basic data collection, the respiratory gas exchange and the blood lactate every minute were simultaneously measured to determine the anaerobic threshold (AT) and the lactate threshold (LT). The results showed that periodical customization of the fuzzy system for individuals was important because of changes in the muscular fatigue properties and the differences between the objective and subjective representations of fatigue [7].

In the investigation of the associations between cardiovascular function and both BMI and PF in Korean men, the VO_2max_ was obtained from YMCA submaximal test using a cycle erogometer. The muscular strength, muscular endurance, flexibility, power, agility, and balance were evaluated by grip strength (kg), sit-ups (reps/min), sit and reach (cm), vertical jump (cm), side steps (reps/30 s), and standing on one leg with eyes closed (s), respectively. This study found that an obese person exhibits lower fitness level and weaker cardiovascular function than a normal person [8].

Tadeusz et al. presented a fuzzy system to evaluate the health related physical fitness (H-RF) based on the European Test of Physical Fitness (EUROFIT) battery tests. The basis of the system is the EUROFIT calculator which converts absolute results of individual trials to standardized values and the fuzzy inference system for four H-RF components (Morphological, cardiorespiratory, musculoskeletal and motor fitness). The system allows one to assess the results of EUROFIT tests in relation to the national reference systems as well as enables their linguistic classification based on the concept of H-RF [9].

The ergoracer bicycle was also used in [10], where a proportional-Integral (PI) fuzzy controller was applied to control the applied physical stress to ensure the predefined target heart rate is not exceeded to ensure safety. The controller accepts two inputs, the first is the error between the subject’s heart rate and the predefined target heart rate (e (t)), while the second is the change of error (Δe (t)). The output is the appropriate physical stress that corresponds to the subject’s current heart rate.

In a similar study [11], physical activity (PA) was assessed by a self-reported questionnaire; cardiorespiratory fitness (CRF) was assessed by VO_2max_ during a symptom-limited maximal exercise test on a cycle ergometer. This study demonstrated that CRF has greater association with the prevalence of metabolic syndrome compared with PA in Chinese midlife women.

A survey to assess cardiorespiratory fitness levels in US youth aged 12 to 19 years was reported in [12]. The estimated VO_2max_ was determined by a submaximal treadmill exercise test. Blood pressure, rating of perceived exertion, and heart rate were obtained during each of the stages. The Jackson non-exercise test formula was used to predict each participant’s fitness level prior to the treadmill test, based on age, sex, BMI, and self-reported physical activity level.

In [13], the cardiovascular system is defined by the following parameters: heart rate (HR, at rest and maximal), stroke volume (SV, at rest and maximal), cardiac output (Q, at rest and maximal), heart volume, blood volume, systolic blood pressure (at rest and maximal), and diastolic blood pressure (at rest and maximal). A fuzzy algorithm evaluates the physiologic effort capacity of an individual based on these physiologic parameters.

A fuzzy PID based on the classic PI control approach was confirmed in a system that included minimizing heart-rate deviations during a treadmill exercise [14]. The controller was tested and validated using two nonlinear human-on-treadmill models [15,16].

The Physical Efficiency Index (PEI), proposed by Brouha et al., is a mathematical formula for determining a cardiovascular endurance index number [17,18]. Lee et al. analyzed the heart rates of Korean children during ski simulator exercise and the Harvard step test to evaluate the cardiopulmonary endurance by the PEI formula. This study showed that the ski simulator exercise can be effectively utilized as exercise equipment since it resulted in higher PEI levels than the Harvard step test [19].

There was another similar survey in Taiwan for health-related physical fitness among junior high school students. Five health-related physical fitness measurements were taken according to the governmental guidelines indicated on the official website of the Taiwan Ministry of Education: body composition (BMI), muscle strength and endurance (bent-leg sit-ups), explosive power (standing long jump), flexibility (sit-and-reach), and cardiorespiratory endurance (800-/1600-m run). All data were processed using Statistical Package for the Social Science (SPSS) software (IBM, Armonk, NY, USA). This study assessed body composition on the basis of BMI. Despite its extensive use to define obesity status, BMI is not an accurate measure of the proportion of fat and fat-free tissue in the body. Percentage of body fat should be considered for precise measures [20].

### 1.2. VO_2max_ and Heart Rate

The heart rate is the most direct response to physiological indicators during quiet rest, exercise and after exercise. Maximal heart rate (MHR) refers to the fastest rate at which your heart will beat in one minute, and is commonly used in exercise physiology and clinical practice for preventive and diagnostic purposes [21]. It is also used to develop exercise prescriptions, estimate aerobic fitness levels, and is often a criterion for achieving maximal exertion in the determination of maximal aerobic capacity. There are different formulas for MHR, in this paper we refer to many ways to provide a more accurate formulas as in Equation (1) to estimate MHR for different age groups [22]:

208 − (0.7 × age)(1)

Resting heart rate is the number of beats per minute of the human heart after a long rest, usually measured in the morning when you wake up and have not yet gotten out of bed. It is normally between 60 (beats per minute) and 100 (beats per minute). RHR can vary with one’s fitness level and with age, and the fitter one is, generally the lower the resting heart rate [23]. This is due to the fact that the heart gets bigger and stronger with exercise, and becomes more efficient at pumping blood around the body, therefore fewer beats per minute are required. Heart rate recovery is the speed at which the heart rate returns to normal after exercise, and it can indicate physical cardiac condition and the risk of certain diseases. Studies often use the current heart rate after stopping the exercise minus the heart rate two minutes after stopping the exercise for measuring HRR, which is a way to tell whether an exercise program is effective. People in better cardiovascular condition tend to have lower heart rates during peak exercise, and return to their resting heart rate more quickly after physical activity [24]. HRR may be an index of physiological age and actual age as illustrated in Table 1.

A person’s VO_2max_ refers to the maximal amount of oxygen the individual can consume typically over one minute during an intense physical effort. The oxygen uptake of the human body is directly proportional to the exercise intensity. Therefore, the oxygen uptake of the human body is an indicator of the level of physical activity. Typically, VO_2max_ is measured directly by analyzing inspired and expired breathing gases in a laboratory setting during maximal exertion, and expressed either as absolute maximal amount of oxygen per minute (L/min) or as relative to the individual’s weight as the maximal milliliters of oxygen the person uses in one minute per kilogram of body weight (mL/kg/min) [25,26,27,28]. The 12-min run fitness test was developed by Cooper in 1968 as an easy way to measure aerobic fitness and provide an estimate of VO_2max_ for military personnel, but today it’s used by many different trainers to determine cardiovascular fitness and track fitness over time [29]. This test requires the athlete to run as far as possible in 12 min, and the distance is measured. However, practice and pacing improve the result, so a swimmer or bicyclist with equal physical fitness would probably score lower. In 2003, the Finnish scholar Saalasti proposed using neural networks for heart rate time series analysis. The cardiopulmonary function instrument was used to collect data of experimenters’ VO_2_ and heart rate (HR). By regression analysis of these data, a nonlinear relationship between VO_2_ and heart rate level is demonstrated together with a polynomial fit to the data. The polynomial can be written as Equation (2) [30]:

(2) $$\dot{V}O_{2}=0.002\times HR^{2}-0.13\times HR+2.3$$

This study found a close relationship between oxygen consumption and heart rate. If laboratory testing is unavailable, then the individual’s age-predicted (estimated) maximal heart rate can be used as the basis for determining exercise intensity. The Karvonen method and percentage of maximal heart rate formulas provide practical intensity assignments basing them on age-predicted maximal heart rates, the relationship between VO_2max_ and MHR is shown in Table 2 [31].

### 1.3. Training and Evaluation

An effective cardiorespiratory endurance training program must include an exercise prescription specifically developed for the individual athlete. It must carefully consider the strengths and weaknesses of the athletes to avoid potentially harmful training programs and cause sports injuries. According to the official guideline of cardiorespiratory endurance of the Sports Administration of Ministry of Education of Taiwan (SAMET), there are four essential components as follows [32]:

Exercise frequency: At least three to five days of aerobic exercise per week.
Exercise intensity: 60–80% of the maximum heart rate is better.
Exercise type: Aerobic exercise is beneficial to the improvement of cardio endurance.
Exercise time: 20–50 min per exercise.

Before exercise, one must first understand one’s physical fitness level; the SAMET physical activity index table helps us to understand our physical condition. The way to do this is to convert the intensity, duration, and frequency of the exercise to a score, and then multiply, that is, the total score of the physical activity index, and then compared the result with Table 3 below, so one can understand one’s physical condition.

Table 4 is the SAMET prescription for aerobic exercise. Users can adjust their exercise load according to their individual physical condition.

Aerobic exercise is beneficial to the improvement of cardiopulmonary fitness. Any aerobic exercise with rhythm, whole body involvement, long time and low intensity such as walking, jogging, aerobic dance, skipping, step exercise, swimming, and bicycle riding can contribute to the improvement of cardiopulmonary fitness. As a result of training, the most obvious physiological changes observed in humans involves their cardiovascular function, respiratory function, neuromuscular system, metabolism, and basic energy systems.

Evaluating these changes is not an easy task. It usually requires expensive equipment and controlled conditions. Besides, using classic training methods often does not ensure the necessary operating points to achieve the desired results, which may cause undesirable events such as under-training or over-training [33]. In recent years, Internet of Things (IoT) technologies have grown rapidly providing an extension of internet connectivity into physical devices and everyday objects. Wearable devices allowing for continuous passive heart rate monitoring have become available [34,35]. The cloud is a huge, interconnected network of powerful servers that performs services for businesses and for people such as Google’s cloud services. Activities like data storage and processing take place in the cloud rather than on the device itself, which has had significant implications for IoT. Many IoT systems make use of large numbers of sensors to collect data and then make intelligent decisions.

In this paper, we propose a fuzzy system based on the human heart rate to provide an effective cardiorespiratory endurance training program and the evaluation of cardiorespiratory endurance levels, so that the trainers can respond correctly to obtain the desired training results and prevent undesirable events. The treadmill is an indoor sport that does not require a wide field and can avoid the effects of weather, and its exercise time and intensity can be controlled, which is suitable for this study. The treadmill training program refers to the 7333 method (exercise at least three times a week, 30 min each time, heartbeat up to 130 bpm). The fuzzy algorithm that is built in the Android mobile phone system receives the resting heart rate (RHR) from a device worn by the participants via Bluetooth before exercise to determine the suitable training speed mode for the individual. The computer-based fuzzy program takes RHR and heart rate recovery (HRR) after exercise as inputs to calculate the cardiorespiratory endurance level. A smart device with a built-in human machine interface (app) receives the training result through the cloud. The proposed fuzzy system not only allows the testers to have better cardio endurance, but also allows the fitness instructor to change the training mode more flexibly by referencing the cardiopulmonary endurance data that is stored in the cloud.

## 2. Materials and Methods

### 2.1. Fuzzy Algorithm

**Figure 1:**
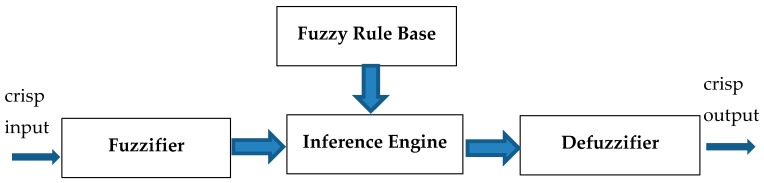
Fuzzy System Diagram.

Intelligent algorithms are a set of computational techniques which try to imitate human brain processing or practices found in Nature to solve a given problem. Their foundations of Nature-inspired computing, such as genetic algorithms, neural networks, evolutionary algorithms and swarm intelligence complements, lie in the evolutionary patterns and behaviors observed in living organisms. The goal of these population-based stochastic optimization techniques is trying to find the optimal problem of the fitness function, which evaluates how well a single solution in a population solves a problem. Complex and uncertain problems usually lack a simple design methodology and their actual implementation is difficult (if not impossible), which cannot be addressed through well-known linear approaches [36]. The fuzzy logic control is an intelligent technique that allows the translation from logic statements to a nonlinear mapping, which is based on rough information for human subjective behavior to quantify operations without constructing complex mathematical functions. Fuzzy systems are rule-based systems, fuzziness in a fuzzy set is characterized by its membership functions that are constructed from a collection of linguistic rules, these rule-based systems in theory model represents any system that work as universal approximators. Fuzzy control presents a way for human thinking to simplify problem complexity. Complexity has always been an important attribute in research methods. The level of qualitative complexity in the fuzzy system increases with the increase of the number of rules. The exact level of this complexity is a function of the overall amount of fuzzy operations during fuzzification, inference and defuzzification, and this amount itself depends on the number of rules [37]. The Fuzzy system can be divided into several parts: fuzzy rule base, membership functions and defuzzification as shown in Figure 1 [38,39,40].

#### 2.1.1. Fuzzifier and Membership Function

**Figure 2:**
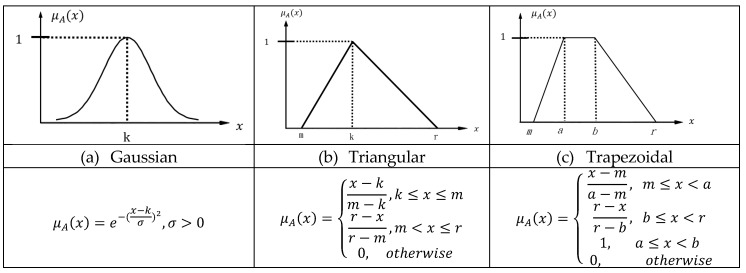
Fuzzy membership functions.

The function of the fuzzifier is to convert the explicit (crisp) external input data into appropriate semantic fuzzy information; that is, to convert the data into fuzzy data. The transformed data will be represented by fuzzy intervals and produce intersections. Each intersection has its own degree of description, the data is then expressed in a colloquial manner after passing through the membership function. It can be seen that the accuracy of the output result is determined according to the number of input parameters given. The fuzzy theory uses the membership function of the numerical region in [0,1] to represent the fuzzy set on the numerical region U. If the function values are all in the interval [0,1], it can be the membership function, such as Gaussian functions, trapezoids, and triangles are the commonly used membership functions in the basic fuzzy inference engine as shown in Figure 2. Trapezoid and triangle membership functions are formed using straight lines, which have the advantage of simplicity. Gaussian membership functions are popular methods for specifying fuzzy sets because of their smoothness and concise notation, but they are unable to specify asymmetric membership functions which are important in certain applications. In specific, the triangular membership function is found to be better than Gaussian membership function [41,42]. In this study, the trapezoid and triangle functions are wide enough to explore the possibilities, and there is not necessary to adopt other expansive membership functions for the proposed fuzzy system.

#### 2.1.2. Fuzzy Rule Database

In general, the fuzzy rule bases are generated according to experts in the particular research field with relevant experience. Fuzzy rules take responsibility for all the classification algorithms in a fuzzy system. The input of fuzzy systems is based on these rules in accordance with the conditions of the fuzzy rules library classification. A determination system controller input of common errors usually has a variation to do error correction. The design approach is based on a fuzzy rule based if-then expressed in the form of a column style. It contains the human judgment of a fuzzy control algorithm expressed in the following Equation (3):

IF [*condition*]*i* THEN [*action*]*i*,   *i* = 1, 2, …, *q* (3)

In which, *q* is the number of rules, the action as performed according to the previous conditions.

#### 2.1.3. Fuzzy Inference Engine

The inference system is the component that derives the fuzzy outputs from the input fuzzy sets according to the relation defined through the fuzzy rules. Rule-based expert systems have the ability to emulate the decision making ability of human experts. They are designed to solve problems as humans do [43], the system can also formulate different inference rules based on human experience. Suppose there are two existing rules as follows: A and B are fuzzy sets, and X and Y are linguistic variables. The fuzzy conditional statement like this one represents a fuzzy relation between A and B defined in UxV. This fuzzy relation is expressed again by a fuzzy set (R) whose membership function is given by Equation (4). $u_{A}\left(x\right)$ and $u_{B}\left(y\right)$ are the membership functions of the fuzzy sets A and B, and I is a fuzzy implication operator that models the existing fuzzy relation [44]. By means of the compositional rule of inference, the fuzzy set B’ is obtained in Equation (5). R is a fuzzy relation defined in U and V, and A’ is a fuzzy set defined in U, the fuzzy set B’ is obtained from the composition of R and A’.

Rule 1.     X is A’
Rule 2.     If X is A, then Y is B
Conclusion   Y is B’

(4) $$\mu_{R}(x,y)=I\left(\mu_{A}\left(x\right),\mu_{B}\left(y\right)\right),\begin{array}{cc} & \forall x\in U,y\in V \end{array}$$

(5) $$B^{\prime }=A^{\prime }\circ R=A^{\prime }\circ (A\to B)$$

Fuzzy relations in different product spaces can be combined with each other by the operation called “Composition” denodes as ‘o’. There are many composition methods in use, e.g., the max-product method, max-average method and max-min method. Among these the max-min composition method is best known in fuzzy logic applications and is used in the system design of this paper. Fuzzy inference use the membership function to obtain the appropriate degree of each rule, and then to synthesize the appropriateness of each rule to get the ideal inference. Equation (6) is the max-min composition of Equation (5):

(6) $$\mu_{B^{\prime }}(y)=\underset{x}{\max}\left[\min\left(\mu_{A^{\prime }}(x),\;\mu_{A\to B}(x,y)\right)\right]$$

#### 2.1.4. Defuzzication

**Figure 3:**
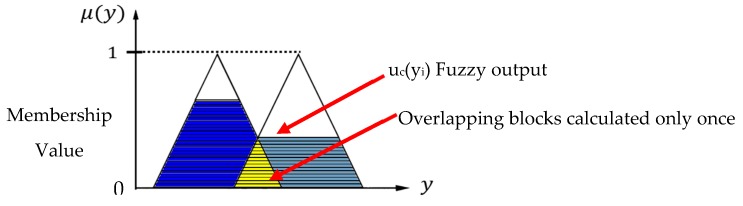
Center of Gravity Method.

“Defuzzification” is the process whereby the fuzzy inference generates conclusions and converts them to a specific value. The Center of Gravity Method (COG) is the most widely used method to solve fuzziness. The output of an entire fuzzy process can be union of two or more fuzzy membership functions as shown in Figure 3. The center of the area generates the center of gravity of the possible distribution of the inferred fuzzy output. It can be defined by the algebraic expression given in Equation (7) [45]:

(7) $$y^{*}=\frac{\sum_{i=1}^{L}\mu_{C}\left(y_{i}\right)\cdot y_{i}}{\sum_{i=1}^{L}\mu_{C}\left(y_{i}\right)}$$

Wherein L is number of the rule base, y* is the output of defuzzification, *y_i_* is the result of the *i*-th fuzzy inferences rule, and *u_c_*(*y_i_*) represents the *y_i_* fuzzy collection of membership values.

### 2.2. System Structure

**Figure 4:**
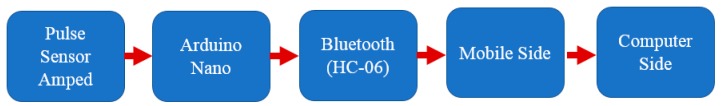
Hardware block diagram.

In order to measure the heartbeat data of the participants, a wearable pulse sensor is used to obtain the statistics of maximum and minimum heartbeat, the minimum value as the resting heart rate and the maximum value as the heartbeat characteristic values during exercise. The Android mobile device displays status messages in real-time. The fuzzy rule base of the training method is designed to get the appropriate speed of the treadmill, and to evaluate cardiorespiratory endurance level after training. The system hardware is based on the Arduino Nano development board [46]. The pulse sensor amped [47] is used to obtain the participants’ exercise heartbeat value, these heartbeat analog signals are sent to the Atmega328 microcontroller chip, and then transmitted to the Android mobile phone via a HC-06 Bluetooth module. The training data is also saved in the SQL database. The hardware block diagram is shown in Figure 4.

#### 2.2.1. System Software Design

**Figure 5:**
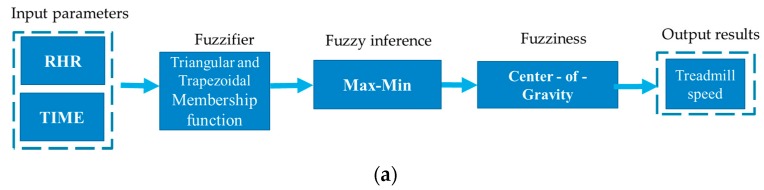

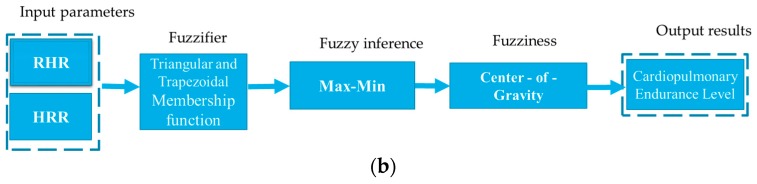
Block diagram of fuzzy inference process (**a**) Block diagram of fuzzy exercise training (**b**) Block diagram of fuzzy cardiorespiratory endurance.

The system software can be divided into the mobile app and computer-based MATLAB desktop environment. MATLAB is an engineering programming language developed by MathWorks (MathWorks, Natick, MA, USA) that expresses matrix and array mathematics. The fuzzy algorithm in the mobile Android app receives the pulse sensing value and calculates the suitable exercise time and speed of the treadmill according to the user’s physical condition. Another fuzzy algorithm on the computer side that applies fuzzy toolbox of MATLAB to evaluate the cardiorespiratory endurance and provide advice. Figure 5 is the block diagrams of the fuzzy process for the mobile Android program and the computer based program for establishing the fuzzy input and output variables, where RHR denotes rest heartrate, TS is treadmill speed, CEL is cardiorespiratory endurance level, HRR denotes heart rate recovery and RHR is the resting heart rate.

Mobile fitness apps have become popular in recent years and are classified as a category called “Fitness Shaping” on Google Play, indicating their popularity in current software development. We obtained some of the more popular fitness apps on Google Play such as Mi Fit [48], Google Fit [49], Nike Run Club [50] and Run on Earth [51] to compare their features with our Android app. The comparison is shown in Table 5. Most of the apps gather users’ sport data and make some statistical analysis for personalize usage. For example, Google Fit uses users’ mobile phone’s sensors or Wear OS on a Google smart watch’s heart rate sensor to record users’ speed, pace, activity route, and more, and gives users one point of cardiopulmonary enhancement score per minute for moderate exercise (such as walking a dog at a faster rate); twice the cardiorespiratory score for higher-intensity activities (such as running). These scores may not reflect the true cardiorespiratory endurance that compare to our study.

#### 2.2.2. Establish Terminology and Membership Functions

**Figure 6:**
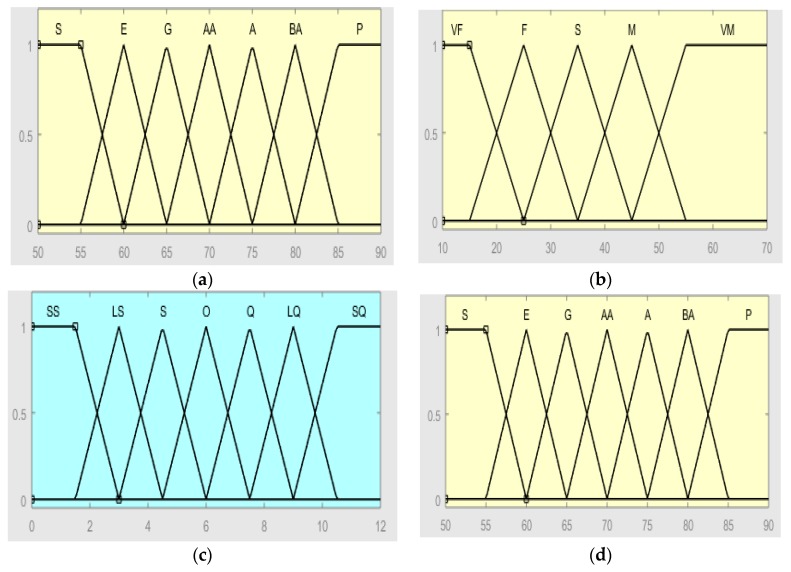

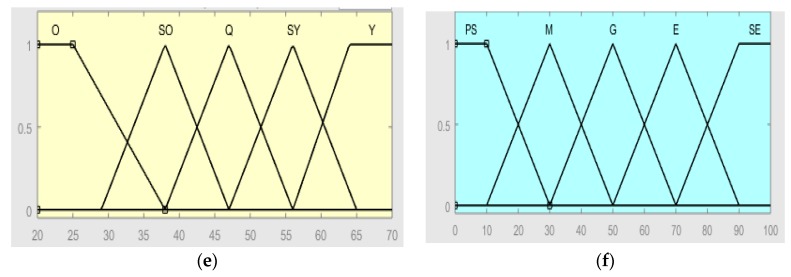
Membership functions (**a**) RHR (**b**) Exercise Time (**c**) TS (**d**) RHR (**e**) HRR (**f**) CEL.

The input parameters RHR (in beats per minute, bpm), Time, HRR (in bpm) terms are divided into P (poor), BA (below average), A (average), AA (above average), G (good), E (excellent), S (sportsman); VF (very few), F (few), S (suitable), M (many), VM (very much); Y (young), SY (slightly young), Q (quite), SO (slightly old) and O (old). The two outputs parameter TS and CEL terms are divided into SS (special slow), LS (little slow), S (slow), O (ordinary), Q (quick), LQ (little quick), SQ (special quick); PS (please strengthen), M (medium), G (good), E (excellent) and SE (special excellent). After the term is established, according to the literature in Section 1, its membership function is defined for each term. The cell-end membership functions are shown in Table 6, Table 7 and Table 8 and Figure 6a–c. The two input variables were “fuzzified”, one of them (RHR) with seven pertinence functions (five triangular and two trapezoidal), another (Time) has five pertinence functions (three triangular and two trapezoidal), and the output has seven pertinence functions (five triangular and two trapezoidal), so 35 rules were defined (7 × 5). The computer-side membership functions are shown in Table 9, Table 10 and Table 11 and Figure 6d–f. The two input variables, one is RHR with seven pertinence functions (five triangular and two trapezoidal), another is HRR with five pertinence functions (three triangular and two trapezoidal), and the output has five pertinence functions (three triangular and two trapezoidal), according to the expert. 

#### 2.2.3. Establish Fuzzy Rules

As discussed in the previous section, Table 1, Table 2, Table 3 and Table 4 are used as the reference for the evaluation of exercise training and cardiorespiratory strength in this system, not only the fuzzy system membership function is defined but it is also used as a reference design for the rule base. The fuzzy rules base of treadmill speed and cardiorespiratory endurance level are illustrated in Table 12 and Table 13. The terminology reference maps are listed in Table 14 and Table 15.

#### 2.2.4. Fuzzy Inference and Defuzzification

**Figure 7:**
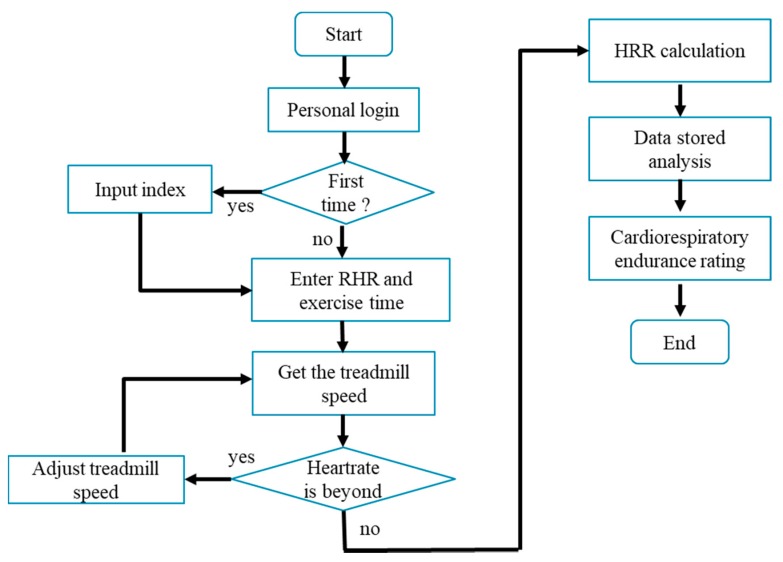
User measurement flow chart.

The hardware sensor measurement system can record all real-time data for calculation, the fuzzy algorithm calculates the treadmill training speed and the cardiopulmonary endurance level through the center of gravity method and show messages to the tester. The user measurement process of this system is shown in Figure 7, which is the human machine interface of the Android app and is mainly divided into three parts: individual login, data access and analysis inference.

**Figure 8:**
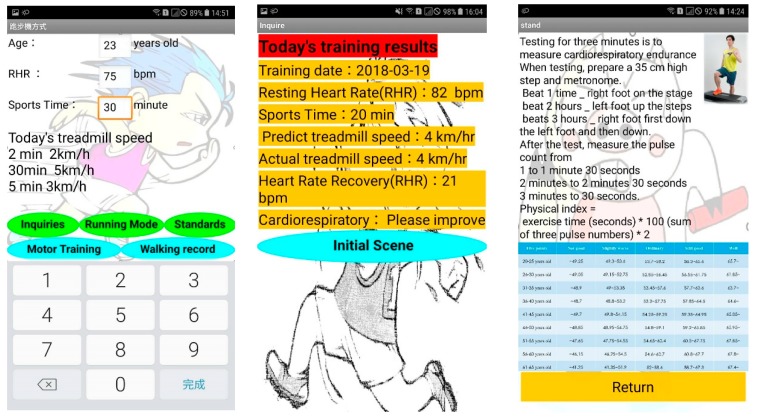
Example of the human machine interface display.

**Figure 9:**
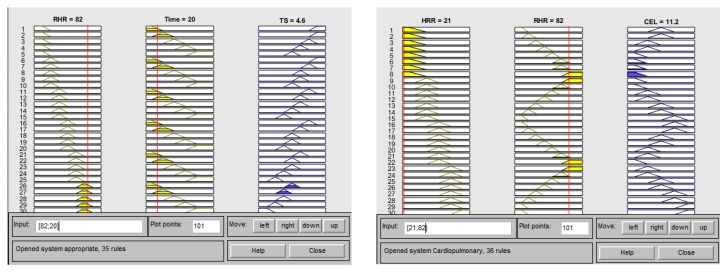
Example of the computer side display.

The testers who are looking for the training mode will first input the basic data for login, and then input RHR and time to get the appropriate treadmill speed. Subjects must wear a lightweight wearable device to monitor the current heart rate, these data will be wirelessly transmitted to the mobile phone system via Bluetooth, and stored in the SQL database through the mobile phone interface. The build-in step meter in the mobile phone allows users to know how many steps they ran. The human machine interface of the proposed cardiorespiratory endurance training and evaluation method is shown in Figure 8, when the RHR is 82 bpm, the exercise time is 20 min, the treadmill speed is 4 km/h, the HRR is 21 bpm after training, and the cardiorespiratory endurance rating is “Please improve”. An example result of the computer side display is shown in Figure 9.

## 3. Experimental Results

### 3.1. Experimental Data

Twenty-five over 18 years old healthy students who regularly exercised (average weekly exercise and occasional weekly exercise) and who volunteered to participate in the experiment for eight weeks were selected. In the experimental process, the participants are number coded from E1 to E25 and divided into two groups. Thirteen people in the control group are responsible for using the system to train, and the reference group used the system and their own ideas to adjust the speed mode for training. The RHR, HRR, mean and standard deviation (STD) of the subjects before and after the experiment are listed in the Table 16. The standard deviation is the square root of the average squared difference between each individual number and the mean of these numbers, which is a number that tells us to what extent a set of numbers lie apart, if a standard deviation is very close to 0, it means that the numbers in a list are almost equal.

### 3.2. Data Analysis

**Figure 10:**
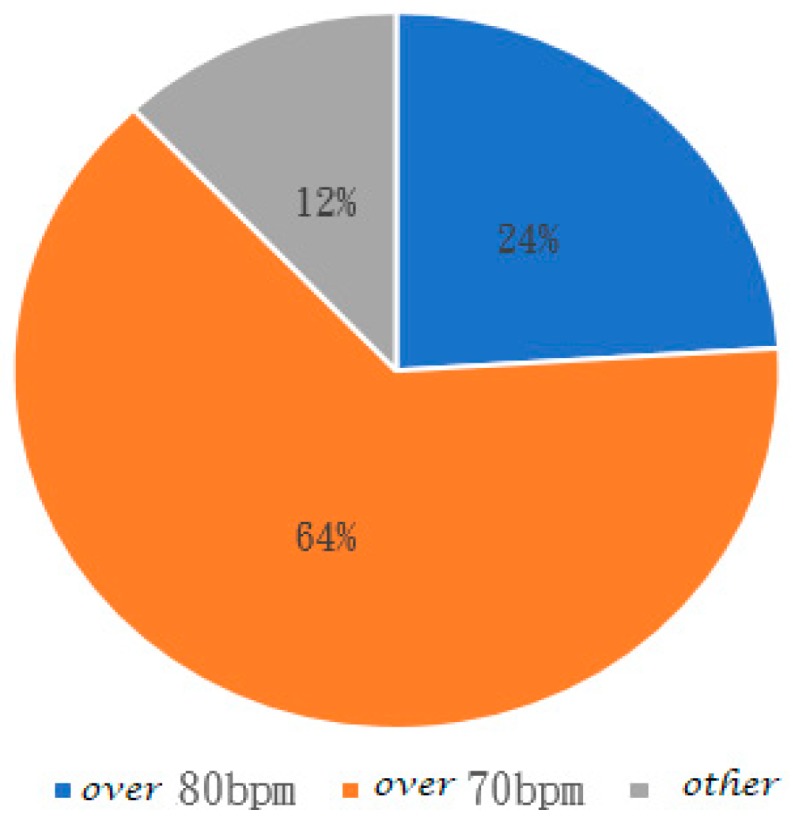
RHR statistic ratio.

Figure 10 shows the statistical ratio by RHR classification of Table 16, where 24% of the participants are over 80 bpm, 64% at 70 bpm, and 12% below 70 bpm.

In this paper two indicators were used for evaluation of cardiorespiratory endurance, which are the decrease rate of RHR (DRHR) and the growth rate of HRR (GHRR) as given in Equations (8) and (9):

DRHR = [(Pre-test RHR) − (Post-test RHR)]/(Pre-test RHR) × 100%(8)

GHRR = [(Post-test HRR) − (Pre-test HRR)]/(Pre-test HRR) × 100%(9)

Subjects in the 80 bpm category are relatively inactive and have a sedentary lifestyle. The ratio of the control group to the reference group is 5:1, they are less likely to adjust the speed of treadmill, so that most of the corresponding cardiorespiratory endurance levels are rated as “Medium”, but DRHR exceeds 10%, and GHRR has a significant growth in Table 17.

Subjects in the 70 bpm category are not completely sedentary in their lives, and sometimes they exercise because of their academic needs, but they may not specifically want to participate in sports for a while. During the training process, more subjects expect that the difficulty of the system can be increased. In the training group, the ratio of the control group to the reference group is 7:9. We can see that there are more “Good” in the cardiorespiratory endurance level, and the DRHR of the reference group is more obvious than that of the control group, but the GHRR was higher in the control group as illustrated in Table 18.

The remaining subjects have the habit of exercising, and they are all classified as “excellent” for cardiorespiratory endurance. In the training group, the ratio of the control group to the reference group is 1:2. During the training process, the subjects generally felt that the system is not suitable for people who regularly exercise. As shown in Table 19, the DRHR has also been improved, but the GHRR was not obvious. This implies that the system is not suitable for people who exercise regularly, because the RHR and HRR of this type are often maintained in a good state, so there is not much space for improvement.

### 3.3. Experimental Result

Table 20 shows the final statistical results of the RHR and HRR of the 25 participants. The post-test value minus the pre-test value is the progress score. The experimental results show that the RHR of the reference group decreased by 7.8 bpm on average, and the HRR increased by 7.9 bpm on average. The RHR of the control group decreased by 8.8 bpm on average, and the HRR increased by 17 bpm on average. The average RHR improvement for all the participants is (11% + 11%)/2 = 11%, and average HRR improvement is (23% + 80%)/2 = 51.5%. After 8 weeks of training, it can be seen clearly in that CEL of the 25 participants has a certain degree of improvement from using the proposed training system.

## 4. Conclusions

Wearable technology, IoT technology and cloud technology are trends of future technological development. This study applies wearable pulse sensors for heart rate measurement, transmission of collected information through IoT and cloud storage, and a multifunctional fitness app with a machine human interface that was built into a smart mobile device for setting the training objective and showing the results. Two different sets of fuzzy algorithms are designed for training and evaluation of cardiorespiratory endurance to help the users develop better exercise habits, so they can use this system to do safe training at home without suffering sport injuries. The experimental results demonstrate the effectiveness of this system in promotion of cardiorespiratory endurance for people without regular exercise habits. The training prescription of this study can make intensity changes for the users with exercise habits, and the training data can support other methods to make the system more perfect and diversified. Aerobic endurance training is a relatively low intensity exercise that depends primarily on aerobic energy generating processes, which is significantly important for maintaining physical activity. Muscle fatigue is one of the main factors that influence sports performance and results in injuries [52]. Combining the fatigue analysis for early warning system into this fuzzy training system could be considered. There are many different factors that affect cardiorespiratory endurance, and the test methods are diverse and complex as mentioned previously, so evaluating these changes is not an easy task. Part of this study is evaluated in an objective way, this will also affect the design of the fuzzy rule base, resulting in inaccurate precision. How to evaluate the cardiorespiratory endurance assessment more accurately with precise and less parameters without requiring expensive equipment and controlled conditions will be studied in our future research.

## Figures and Tables

**Table 1:** Relationship between HRR, physiological age and actual age.

HRR	Evaluation
<22	physiological age is older than the actual age
22–52	physiological age is equivalent to the actual age
53–58	physiological age is slightly younger than the actual age
59–65	physiological age is younger than the actual age
≥66	physiological age is much younger than the actual age

**Table 2:** Relationship between VO_2max_, HRR, and MHR.

**% VO_2max_**	50	55	60	65	70	75	80	85	90	95	100
**% MHR**	66	70	74	77	81	85	88	92	96	98	100

**Table 3:** Cardiopulmonary endurance evaluation (total score = intensity × time × frequency).

Total Score	Assessment	Category
>100	Very active lifestyle	Very good
80–100	Active and healthy	Good
40–80	Acceptable but can be better	Normal
20–40	Insufficient exercise	Bad
<20	Static lifestyle	Very Bad

**Table 4:** Aerobic exercise prescription.

Stage	Begin		Improvement								Maintain
Week	1–2	3–4	5–6	7–8	9–10	11–12	13–14	15–16	17–18	19–20	>20
Frequency/Week	3	3	3	3	3	3	4	4	4	4	5
Duration (min)											
Warm up	5	5	5	6	6	7	7	8	8	8	8
Main	10	13	15	15	20	20	23	25	28	30	>35
Cool down	20	23	25	27	32	34	37	40	43	45	7
Total time (min)	20	23	25	27	32	34	37	40	43	45	>50
Strength (MHR %)	55	55	60	60	65	65	65	70	70	70	75

**Table 5:** Feature comparisons of fitness apps.

Feature App	Pedometer (Step Counter)	Sports Data Collection	Statistical Analysis	Personalized	Additional Sensor	Fitness Equipment Combined	Sports Planning
Mi Fit	O	O	O	O	O	X	X
Google Fit	O	O	O	O	O	X	X
Nike Run Club	O	O	O	O	X	X	O
Run on Earth	X	X	O	O	X	O	O
This study	O	O	O	O	O	O	O

‘O’ denotes ‘support’, ‘X’ denotes ‘not support’.

**Table 6:** RHR membership function.

RHR	P	BA	A	AA	G	E	S
bpm	80–90	75–85	70–80	65–75	60–70	55–65	50–60

**Table 7:** Time membership function.

Time	VF	F	S	M	VM
min	10–25	15–35	25–45	35–55	45–70

**Table 8:** TS membership function.

TS	SS	LS	S	O	Q	LQ	SQ
Km/h	0–3	1.5–4.5	3–6	4.5–7.5	6–9	7.5–10.5	9–12

**Table 9:** RHR membership function.

RHR	P	BA	A	AA	G	E	S
bpm	80–90	75–85	70–80	65–75	60–70	55–65	50–60

**Table 10:** HRR membership function.

HRR	O	SO	Q	SY	Y
bpm	20–38	29–47	38–56	47–65	55–70

**Table 11:** CEL membership function.

CEL	PS	M	G	E	SE
level	0–30	10–50	30–70	50-90	70–100

**Table 12:** Fuzzy rules base of treadmill speed.

	Input1		Input2		Output
If	RHR is Sportsman	and	Time is Very Few	Then	TS is Special Quick
If	RHR is Sportsman	and	Time is Few	Then	TS is Special Quick
If	RHR is Sportsman	and	Time is Suitable	Then	TS is Little Quick
If	RHR is Sportsman	and	Time is Many	Then	TS is Quick
If	RHR is Sportsman	and	Time is Very Much	Then	TS is Ordinary
If	RHR is Excellent	and	Time is Very Few	Then	TS is Special Quick
If	RHR is Excellent	and	Time is Few	Then	TS is Little Quick
If	RHR is Excellent	and	Time is Suitable	Then	TS is Quick
If	RHR is Excellent	and	Time is Many	Then	TS is Quick
If	RHR is Excellent	and	Time is Very Much	Then	TS is Ordinary
If	RHR is Good	and	Time is Very Few	Then	TS is Little Quick
If	RHR is Good	and	Time is Few	Then	TS is Quick
If	RHR is Good	and	Time is Suitable	Then	TS is Quick
If	RHR is Good	and	Time is Many	Then	TS is Ordinary
If	RHR is Good	and	Time is Very Much	Then	TS is Slow
If	RHR is Above Average	and	Time is Very Few	Then	TS is Little Quick
If	RHR is Above Average	and	Time is Few	Then	TS is Quick
If	RHR is Above Average	and	Time is Suitable	Then	TS is Ordinary
If	RHR is Above Average	and	Time is Many	Then	TS is Ordinary
If	RHR is Above Average	and	Time is Very Much	Then	TS is Slow
If	RHR is Average	and	Time is Very Few	Then	TS is Quick
If	RHR is Average	and	Time is Few	Then	TS is Ordinary
If	RHR is Average	and	Time is Suitable	Then	TS is Ordinary
If	RHR is Average	and	Time is Many	Then	TS is Slow
If	RHR is Average	and	Time is Very Much	Then	TS is Little Slow
If	RHR is Below Average	and	Time is Very Few	Then	TS is Ordinary
If	RHR is Below Average	and	Time is Few	Then	TS is Slow
If	RHR is Below Average	and	Time is Suitable	Then	TS is Slow
If	RHR is Below Average	and	Time is Many	Then	TS is Little Slow
If	RHR is Below Average	and	Time is Very Much	Then	TS is Special Slow
If	RHR is Poor	and	Time is Very Few	Then	TS is Slow
If	RHR is Poor	and	Time is Few	Then	TS is Little Slow
If	RHR is Poor	and	Time is Suitable	Then	TS is Little Slow
If	RHR is Poor	and	Time is Many	Then	TS is Special Slow
If	RHR is Poor	and	Time is Very Much	Then	TS is Special Slow

**Table 13:** Fuzzy rules base of cardiorespiratory endurance level.

	Input1		Input2		Output
If	HRR is Old	and	RHR is Sportsman	Then	CEL is Good
If	HRR is Old	and	RHR is Excellent	Then	CEL is Good
If	HRR is Old	and	RHR is Good	Then	CEL is Medium
If	HRR is Old	and	RHR is Above Average	Then	CEL is Medium
If	HRR is Old	and	RHR is Average	Then	CEL is Please Strengthen
If	HRR is Old	and	RHR is Below Average	Then	CEL is Please Strengthen
If	HRR is Old	and	RHR is Poor	Then	CEL is Please Strengthen
If	HRR is Slightly Old	and	RHR is Sportsman	Then	CEL is Excellent
If	HRR is Slightly Old	and	RHR is Excellent	Then	CEL is Good
If	HRR is Slightly Old	and	RHR is Good	Then	CEL is Good
If	HRR is Slightly Old	and	RHR is Above Average	Then	CEL is Medium
If	HRR is Slightly Old	and	RHR is Average	Then	CEL is Medium
If	HRR is Slightly Old	and	RHR is Below Average	Then	CEL is Please Strengthen
If	HRR is Slightly Old	and	RHR is Poor	Then	CEL is Please Strengthen
If	HRR is Quite	and	RHR is Sportsman	Then	CEL is Excellent
If	HRR is Quite	and	RHR is Excellent	Then	CEL is Excellent
If	HRR is Quite	and	RHR is Good	Then	CEL is Good
If	HRR is Quite	and	RHR is Above Average	Then	CEL is Good
If	HRR is Quite	and	RHR is Average	Then	CEL is Medium
If	HRR is Quite	and	RHR is Below Average	Then	CEL is Medium
If	HRR is Quite	and	RHR is Poor	Then	CEL is Please Strengthen
If	HRR is Slightly Young	and	RHR is Sportsman	Then	CEL is Special Excellent
If	HRR is Slightly Young	and	RHR is Excellent	Then	CEL is Excellent
If	HRR is Slightly Young	and	RHR is Good	Then	CEL is Excellent
If	HRR is Slightly Young	and	RHR is Above Average	Then	CEL is Good
If	HRR is Slightly Young	and	RHR is Average	Then	CEL is Good
If	HRR is Slightly Young	and	RHR is Below Average	Then	CEL is Medium
If	HRR is Slightly Young	and	RHR is Poor	Then	CEL is Medium
If	HRR is Young	and	RHR is Sportsman	Then	CEL is Special Excellent
If	HRR is Young	and	RHR is Excellent	Then	CEL is Special Excellent
If	HRR is Young	and	RHR is Good	Then	CEL is Excellent
If	HRR is Young	and	RHR is Above Average	Then	CEL is Excellent
If	HRR is Young	and	RHR is Average	Then	CEL is Good
If	HRR is Young	and	RHR is Below Average	Then	CEL is Good
If	HRR is Young	and	RHR is Poor	Then	CEL is Medium

**Table 14:** Reference of treadmill speed.

Treadmill Speed (km/h)		RHR (BPM)						
		P	BA	A	AA	G	E	S
Time (Min)	VF	S	O	Q	LQ	LQ	SQ	SQ
	F	LS	S	O	Q	Q	LQ	SQ
	S	LS	S	O	O	Q	Q	LQ
	M	SS	LS	S	O	O	Q	Q
	VM	SS	SS	LS	LS	S	O	O

**Table 15:** Reference of cardiorespiratory endurance level.

CEL		RHR (BPM)						
		P	BA	A	AA	G	E	S
HRR (BPM)	O	PS	PS	PS	M	M	G	G
	SO	PS	PS	M	M	G	G	E
	Q	PS	M	M	G	G	E	E
	SY	M	M	G	G	E	E	SE
	Y	M	G	G	E	E	SE	SE

**Table 16:** Experimental Data.

Reference Group				
Code Number	Pre-Test		Post-Test	
	RHR (BPM)	HRR (BPM)	RHR (BPM)	HRR (BPM)
E2	76	32	68	45
E3	75	30	67	42
E4	76	32	68	44
E6	71	42	60	50
E11	60	46	56	51
E12	58	52	55	52
E13	80	33	66	42
E16	70	38	67	40
E17	74	38	65	43
E20	70	42	65	48
E24	76	32	66	43
E25	79	31	69	42
Mean & STD	72.1 ± 6.6	37.3 ± 6.7	64.3 ± 4.5	45.2 ± 3.9
**Control Group**				
E1	82	21	71	40
E5	76	22	71	39
E7	80	21	67	45
E8	75	25	71	38
E9	78	20	71	33
E10	82	18	70	37
E14	78	18	72	34
E15	75	22	71	36
E18	68	48	58	56
E19	79	25	70	41
E21	81	20	68	40
E22	79	21	69	42
E23	81	20	70	41
Mean & STD	78 ± 3.7	23.2 ± 7.5	69.2 ± 3.5	40.2 ± 5.6

**Table 17:** RHR over 80 bpm Category.

Reference Group					
NO.	DRHR (%)	Time (min)	TS (km/h)	GHRR (%)	CEL
E13	18%	50	4.7	27%	M
**Control Group**					
E1	13%	34.55	4.5	90%	M
E7	16%	35.42	5.4	114%	G
E10	15%	40	4.6	106%	M
E21	16%	51.6	3.3	100%	M
E23	14%	40.4	4.5	105%	M

**Table 18:** RHR 70–80 bpm Category.

Reference Group					
NO.	DRHR (%)	Time (min)	TS (km/h)	GHRR (%)	CEL
E2	11%	42.92	5	41%	G
E3	11%	43.04	5.3	40%	M
E4	11%	35.45	6.4	38%	G
E6	15%	47.71	6.1	19%	G
E16	4%	32.5	5.9	5%	M
E17	12%	42.92	5	13%	G
E20	7%	43.91	6.1	14%	G
E24	13%	31.67	5.6	34%	G
E25	13%	46.36	4.8	35%	M
**Control Group**					
E5	7%	32.5	5.3	77%	M
E8	5%	38.33	5	52%	M
E9	9%	41.67	4.4	65%	M
E14	8%	45	4	89%	M
E15	5%	37.2	5.3	64%	M
E19	11%	39.13	5.1	64%	G
E22	13%	38.8	4.7	100%	M

**Table 19:** RHR below 70 bpm Category.

Reference Group					
NO.	DRHR (%)	Time (min)	TS (km/h)	GHRR (%)	CEL
E11	7%	32.5	7.6	11%	E
E12	5%	32.5	7.8	0%	SE
**Control Group**					
E18	15%	34.35	7.2	17%	E

**Table 20:** RHR & HRR Statistical Results.

**Reference Group**							
**Item**	**Pre-Test**		**Post-Test**		**(Post-Test)-(Pre-Test)**		**Average Improvement (%)**
	**Mean**	**STD**	**Mean**	**STD**	**Mean**	**STD**	
RHR	72.1	6.6	64.3	4.5	−7.8	2.1	11%
HRR	37.3	6.7	45.2	3.9	7.9	2.8	23%
**Control Group**							
**Item**	**Pre-Test**		**Post-Test**		**(Post-Test)-(Pre-Test)**		**Average Improvement (%)**
	**Mean**	**STD**	**Mean**	**STD**	**Mean**	**STD**	
RHR	78	3.7	69.2	3.5	−8.8	0.2	11%
HRR	23.2	7.5	40.2	5.6	17	1.1	80%
